# Preconception nutraceutical food supplementation can prevent oxidative and epigenetic DNA alterations induced by ovarian stimulation for IVF and increases pregnancy rates

**Published:** 2020-05-07

**Authors:** W Decleer, F Comhaire, K De Clerck, W Vanden Berghe, G Devriendt, K Osmanagaoglu

**Affiliations:** Fertility-Belgium Clinic, Weststraat, 16-18, B-9880 Aalter, Belgium;; Lab Protein Science, Proteomics and Epigenetic Signaling, Department of Biomedical Sciences, University Antwerp, Campus Drie Eiken, Universiteitsplein 1, B-2610 Wilrijk, Belgium;; Pures Ltd., Kasteelhoek 12, B-8730 Beernem, Belgium;; Centre for Fertility, AZ Jan Palfijn Gent, Watersportlaan 5, B-9000 Gent, Belgium.

**Keywords:** Epigenetics, hTERT promoter, 8-hydroxy-2-deoxyguanosine, nutraceuticals, in vitro fertilisation, infertility

## Abstract

**Background:**

It is hypothesized that oxidative and epigenetic alterations to DNA induced by ovarian stimulation for in vitro fertilization (IVF) may be associated with an increased risk of diseases and cancer in the offspring and could possibly be attenuated by preconception food supplementation.

**Methods:**

In a prospective randomised open-label trial, 62 patients were randomly assigned to either 30 days of preconception treatment with the nutraceutical Fertility woman ® duo (Nutriphyt, Beernem, Belgium) (group 1), this nutraceutical complemented with selenomethionine (group 2), or folic acid only (group 3). Biochemical and epigenetic effects and pregnancy rates were assessed.

**Results:**

In all 3 groups the level of DNA oxidative damage, estimated by the concentration of 8-hydroxy- 2-deoxyguanosine over creatinine in early morning urine, and the concentration of homocysteine in the blood decreased after treatment. In group 2, the degree of methylation of the cancer-associated CpG2 dinucleotide of the human Telomerase Reverse Transcriptase (hTERT) promoter region, assessed by pyrosequence in follicular cells obtained at oocyte pick-up, was 18% lower than that of group 3. The pregnancy rate, including the transfer of fresh and frozen embryos, was significantly higher in group 2 (50%) than in group 3 (6%) with the result in group 1 being intermediate (30%).

**Conclusion:**

The results suggest that preconception food supplementation using a specific nutraceutical significantly reduces oxidative and epigenetic DNA changes to follicular cells of women treated by IVF, and may optimize gene expression in the oocytes, thus increasing the pregnancy rate per cycle of ovarian stimulation.

## Introduction

Since the birth of the first in vitro fertilization (IVF) baby 4 decades ago, this technique has generated several millions of offspring for infertile couples worldwide. Two important problems still remain unresolved, namely the relatively low pregnancy rate per initiated cycle and a possible adverse effect on the offspring (Gleicher and Barad, 2019). The occurrence of epigenetic changes, with elevated methylation of particular dinucleotides of the human telomerase reverse transcriptase (hTERT) promoter region in follicular cells after controlled ovarian stimulation has been reported (Comhaire et al., 2015). The latter may possibly be related to the alleged increase in the prevalence of certain cancers in children born after IVF (Moll et al., 2003; Källén et al., 2010; Petridou et al., 2012; Castelo-Branco et al., 2013; Reigstad et al., 2016; Wainstock et al., 2017; Spaan et al., 2019; Spector et al., 2019), as well as metabolic and cardiovascular diseases (Scherrer et al., 2015) occurring more frequently in those children (Hwang et al., 2018).

Epigenetic DNA-changes (Chen and Heilbronn et al., 2017), abnormal methylation in particular, depend on external factors including the nutritional supply of vitamins and minerals. Oxidative damage of DNA causing increased transition mutagenesis results from an elevated concentration of 8-hydroxy- 2-deoxy guanosine (8OH-2dG).

Ovarian stimulation for IVF has been reported to cause increased concentrations of homocysteine in blood and in follicular fluid (Pacchiarotti et al., 2007; Boxmeer et al., 2009) that, among other things, is associated with poor embryo quality and with unfavourable pregnancy outcomes (Berker et al., 2009; Ocal et al., 2012). Elevated homocysteine may influence DNA methylation (Mandaviva et al., 2014), particularly at the CpG sites. It should be stressed that homocysteine may either decrease or increase DNA methylation through the one carbon pathway. Aside from the effect of oestrogens, external factors, nutrition in particular (Alshatwi and Shafi, 2012), influence the balance between the two main cofactors S-adenosylmethionine (SAM) and S-adenosylhomocysteine (SAH) in transferring methyl groups to the DNA. These factors can be corrected by means of the complementary intake of appropriate food supplements (Comhaire et al., 2017).

In the present prospective randomised and controlled trial (Mathes et al., 2018; Mathes et al., 2019), we have studied the effect of different substances given for food supplementation on several biological and (epi-)genetic markers that may serve as a surrogate for possible adverse effects in IVF-offspring and on pregnancy rates (Oostingh et al., 2019).

## Materials and methods

Sixty-two infertile couples, programmed for IVF, were invited to participate in a randomised prospective trial comparing the effect of food supplementation given to the women on biological variables, including epigenetic DNA-changes, and on the pregnancy rate. The ethical committee of the Jan Palfijn Hospital, Ghent, Belgium, approved the research and patients gave informed consent to participate in the project (2017/009).

One month before the planned date of the oocyte pick-up, blood was taken and early morning urine was collected (Poulsen et al., 2014). The women were randomly assigned to take either the nutraceutical “Fertility woman ® ” (Nutriphyt, Beernem, Belgium, for composition see appendix) (Comhaire and Mahmoud, 2013) (group 1), “Fertility woman ® ” plus Selenomethionine (70 μg Se) (group 2), or folic acid (0.4 mg vitamin B9) (group 3, control group) until the day of oocyte pick-up. For the statistics on DNA methylation, the results of the epigenetic analysis performed on follicular fluid cells of 8 untreated cases reported in a previous study were pooled with group 3 data (Comhaire et al., 2015). On the day of pick-up, blood was taken again and urine was collected. During oocyte pick-up follicular fluid was collected for analysis. Randomization was performed by the project coordinator using a computer program (GraphPad.com/QuickCalc/randomize1).

All testing methods were fully validated and submitted to continuous internal and external quality assessment (Table I).

**Table I:** Biological measurements performed.

Biological measurements	Reference values
8-hydroxy-2-deoxyguanosine, 8OH-2dG RP lab, Synlab Brussels, Soldatenstraat, 40, 1082 Sint-Agatha-Berchem, Belgium	2-20 μg/l
quotient of 8OH-2dG divided by the concentration of creatinine (early morning urine) RP lab, Synlab Brussels, Soldatenstraat, 40, 1082 Sint-Agatha-Berchem, Belgium	0-35 μg/g creatinine
Vitamin B12 (serum) Labo Jan Palfijn Hospital, Henri Dunantlaan, 5, 9000 Gent, Belgium	>145 pg/mL
Folic acid (serum) Labo Jan Palfijn Hospital, Henri Dunantlaan, 5, 9000 Gent, Belgium	>4 ng/mL
Homocysteine (serum) AML, Emiel Vloorsstraat 9, 2020 Antwerpen, Belgium	<12 μmol/L
Estradiol (serum) Labo Jan Palfijn Hospital, Henri Dunantlaan, 5, 9000 Gent, Belgium	Follicular fase: 26.6 - 161 ng/L Peri-ovulatory fase: 187 – 382 ng/L Luteal fase: 32.7 – 201ng/L

Patients were stimulated to develop several follicles according to conventional methods, using human menopausal gonadotropin (hMG, Menopur ® , Ferring Pharmaceuticals, Saint Prex, Switzerland) three ampules of 75 IU subcutaneously daily until a follicular diameter of at least 18 mm was attained. Oocyte maturation was triggered with 5000 IU of human chorionic gonadotropin (hCG, Pregnyl, MSD, Kenilworth, New Jersey, United Sates), injected 36 hours before oocyte pick-up. Aspiration of the follicular fluid was performed through a Cook 17-gauge aspiration needle (K-OSN-1730-B-60) with a negative pressure of 130 mmHG (Cook vacuum pump UL 60601-1, CAN/CSA-C22.2). Prior to follicular puncture, the needle was rinsed with 37°C physiological Hartmann Viaflo isotonic solution (Baxter, USA). The cumulus oocyte complexes were isolated in Human Tubal Fluid medium (HTF, IVF Basics, Gynotec, Netherlands) with supplemental Human Albumin and transferred into a culture medium (Sequential medium G1 Vitrolife, Sweden or Cleavage medium Origio, Netherlands, Cooper Surgical, USA).

Fertilisation was achieved by Intracytoplasmatic Sperm Injection. Embryo transfer was performed on day 3 after fertilisation using the Cook Guardia catheter complex. Good quality spare embryos were snap frozen by vitrification on day 5 or 6 on the condition that they developed at least into blastocyst- one stage with a clear notification of the inner cell mass (Fast Freeze, Life Global, Cooper Surgical, USA). Frozen/thawed embryos were transferred in the subsequent or later cycles, preferably -if the patient had a regular cycle- in a natural cycle, 5 days after spontaneous LH surge. If the patient did not have a natural cycle, an artificial cycle was created using Progynova ® , Utrogestan ® and, if necessary, intramuscular progesterone administration.

The thawing process (prior to the transfer) was performed the morning of the day of transfer.

The remaining follicular fluid -on the condition that it was macroscopically free of blood- of preferentially 2 follicles of each ovary was collected, immediately pelleted by centrifugation (3000 rpm, 10 min) and aspirated into straws. These were snap frozen and transported on dry ice and stored at minus 70°C until analysis. Genomic DNA was subsequently isolated from frozen pellets with DNeasy blood and tissue kit (Qiagen, Antwerp, Belgium).

Epigenetic analysis for the measurement of the degree of methylation of the hTERT promotor and its five individual dinucleotides was performed almost exactly as described by Castelo-Branco et al. (2013) with minor modifications. In short, 1 μg gDNA of each sample was sodium bisulfite converted using the EpiTect fast DNA bisulfite kit (Qiagen) according to manufacturer’s instructions. Fifteen ng bisulfite converted DNA was used as an input for PCR amplification using the PyroMark PCR kit (Qiagen). Finally, pyrosequencing was performed using the PyroMark Q24 instrument and PyroMark Q24 advanced CpG reagents (Qiagen). Methylation percentages for each CpG were obtained and analysed using the PyroMark Q24 advanced software (Qiagen), which calculates the methylation percentage (mC/(mC+C)) for each CpG site, allowing for quantitative comparison. Only samples that passed quality control after pyrosequencing were used for further analysis and graphical representation. The hTERT pyrosequencing assay was designed using the PyroMark assay design software (Qiagen). Following primers were used: forward ATGTGGAGGTTTTGGGAATAG, reverse AACCTAAAAACAACCCTAAATCT and sequencing GGTTTTGGGAATAGGTG, spanning the hTERT promoter region (chr5:1,295,586- 1,295,643, GRCh37/hg19). All methylation analysis was performed by technical staff who did not have any information regarding tissue diagnosis or patient outcomes.

Simultaneously positive reference gDNA material isolated from highly metastatic MDA-MB231 breast cancer cell lines was analysed for cancer- related DNA-hyper-methylation, to serve as internal positive validation of the epigenetic test procedure.

Since it seems ethically unacceptable or technically unfeasible to aspirate follicular fluid in healthy women not treated by assisted reproduction, the experimental setup does not include follicular fluid of untreated controls. gDNA-depleted samples served as negative test controls and did not yield any signal.

The following demographic and biological data were also recorded: age, body mass index (BMI), number of previous IVF trials (cycle number), number of oocytes retrieved, and hCG concentration at 14 days after embryo transfer. Concentrations of hCG in excess of 6 mU/mL were considered indicative of pregnancy.

The results were plotted in an Excel file and transferred into the MedCalc ® statistical program (MedCalc, Ostend, Belgium) (Schoonjans et al., 1995). Parametrical and non-parametrical statistical tests were performed as required, as well as receiver operating characteristic (ROC) curve analysis (Schoonjans et al., 1996) and multiple and logistic regression analyses with stepwise elimination. Among the CpG2 measurements, one outlier value exceeding 33% methylation occurred in each group. These values, i.e. 3 out of 216 observations, were excluded from statistical analysis (Grubbs, 1969).

Calculation of the number of pregnancies was done separately for those occurring after fresh transfer, for those after transfer of frozen embryos (frozen transfer), and for the total number of pregnancies following one single stimulation with oocyte pick-up procedure, adding up the fresh and frozen transfers.

The number needed to treat (NNT) was calculated by dividing 100 by the percentage difference between the pregnancy rates in the intervention group (either group 1 or group 2) minus the pregnancy rate in the control group (group 3).

## Results

### 

Table II displays the number of patients enrolled and those who completed the study, the number of pregnancies and the proportion of pregnancies per group.

**Table II:** Number of patients enrolled, number of cases who completed the study, number of pregnancies and proportion of pregnancies (in percent) per group and in all cases pooled.

	Group 1	Group 2	Group 3	All cases
Number of cases recruited	23	21	18	62
Number of cases completed	20	20	17	57
Number pregnant following fresh transfer	3	6	1	10
% pregnant	15	30	5,9	17,5
Number pregnant following frozen transfer	3	5	0	8
Number pregnant fresh plus frozen	6	11	1	18
% pregnant following fresh plus frozen transfer (per recruited)	26	53	6	29
% pregnant following fresh plus frozen transfer (per pick-up)	30	55	6	32

Out of 62 couples enrolled, 58 were randomised. Four were not randomised: two because of moving abroad, one because spontaneous pregnancy occurred during intake of the supplement (group 1), and one because IVF had to be converted to IUI for financial reasons. In one patient, no oocytes could be retrieved, and no transfer took place. Hence, there was embryo transfer in 57 cases.

Ten pregnancies occurred after fresh transfer, 3 in group 1, 6 in group 2 and 1 in group 3. After frozen transfer, 8 pregnancies occurred of which 3 were in group 1 and 5 were in group 2. Thus, 18 out of 57 transfers resulted in a positive pregnancy test (hCG >6 IU/L) corresponding to 31.6%.

Table III lists the demographic characteristics, including age, BMI, number of previous cycles, and the results of the biological measurements before and after treatment of the entire population, and separately per group.

**Table III:** Demographic and clinical data and values of variables studies (means and standard deviations, SD) before and after nutraceutical intake in the entire population and the groups. Significant changes between before and after are printed in bold.

	Group 1	Group 2	Group 3	All cases
Age (years)	32,26 (5,4)	31,38 (4,81)	32,11 (4,89)	31,92 (4,99)
Number of cases	23	21	18	62
BMI (kg/m^2^)	24,61 (5,97)	23,11 (4,3)	25,09 (6,19)	24,24 (5,5)
Cycle number	2,39 (1,27)	2,5 (1,47)	2,5 (2,38)	2,46 (1,7)
8OH-2dG/creat before (μg/l/μg/g)	**10,85 (6,39)**	**10,79 (4,83)**	10,68 (4,48)	**10,78 (5,29)**
8OH-2dG/creat after (μg/l/μg/g)	**8,61 (3,85)**	**8,2 (5,41)**	8,25 (5,16)	**8,87 (4,8)**
Homocystein before (μmol/l)	6,64 (2,16)	**7,93 (4,71)**	**7,73 (2,75)**	**7,41 (3,38)**
Homocystein after (μmol/l)	5,8 (1,4)	**6,64 (3,0)**	**6,63 (2,66)**	**6,32 (2,4)**
Folic acid before (ng/ml)	**14,1 (6,86)**	**12,43 (7,11)**	12,97 (7,53)	**13,21 (7,06)**
Folic acid after (ng/ml)	**16,22 (5,59)**	**15,08 (6,69)**	13,89 (7,34)	**15,15 (6,56)**
Vitamin B12 before (pg/ml)	293 (115)	305,7 (164,6)	336,1 (305,1)	209,8 (199,8)
Vitamin B12 after (pg/ml)	329,7 (141,6)	319,1 (117)	306,2 (251,5)	319,2 (171,8)

### Comparison of concentrations before and after supplement intake.

The ratio of 8OH-2dG/creatinine decreased in the entire study population (Wilcoxon rank sum test for paired observation, p=0.005), significantly in groups 1 and 2 (p<0.05), but not significantly in group 3 (p=0.065) (Table III). The concentration of homocysteine decreased in the entire study population (p=0.0001), significantly in groups 2 (p=0.013) and 3 (p=0.011), but not significantly in group 1 (p=0.089). The concentration of vitamin B9 increased in the entire study population (p=0.001), significantly so in groups 1 (p=0.026) and 2 (p=0.015), but not significantly in group 3 (p=0.389). The concentration of vitamin B12 did not change significantly with p=0.549 for the entire study population, nor in each group separately (p>0.20).

### Correlations between variables

The following correlations (Table IV) were highly significant (p<0.01): vitamin B9 (r=0.85), B12 (r=0.88), and homocysteine (r=0.87) before and after food supplementation, homocysteine versus vitamin B12 (r=-0.40 and r=-0.44 before and after food supplementation respectively), homocysteine versus vitamin B9 (r=-0.54 and r=-0.64 before and after supplementation) and vitamin B9 versus vitamin B12 (r=0.44 and r=0.49 before and after supplementation). The number of oocytes retrieved was highly significantly correlated with the maximum estradiol concentration (r=0.68, p<0.0001) and with the cycle number (r=0.40, p=0.002).

**Table IV:** Cross tabulation of correlations between variables recorded in this trial (student t-test: r= correlation coefficient, followed by P value). Significant correlations are printed in bold. Legend to units see table II.

	BMI	cycle number	number oocyte	max estradiol	vit B12 before	vit B12 after	8OH2dG/cr before	8OH2dG/cr after	vit B9 before	vit B9 after	homocyst before	homocyst after
age	r=0,02 0,90	r=0,00 0,96	**r=-0,32 0,01**	**r=-0,27 0,04**	r=0,04 0,74	r=-0,04 0,75	r=-0,04 0,76	r=0,06 0,67	r=0,19 0,13	r=0,20 0,14	r=0,00 0,99	r=-0,04 0,76
BMI		r=-0,15 0,23	r=-0,15 0,26	r=-0,08 0,53	r=-0,17 0,19	r=-0,17 0,20	r=-0,02 0,87	r=-0,13 0,32	r=-0,17 0,19	r=-0,02 0,89	r=0,05 0,72	r=-0,04 0,76
cycle number			**r=0,40 0,002**	**r=0,31 0,02**	r=0,20 0,11	r=0,16 0,22	r=-0,12 0,37	r=-0,02 0,90	r=0,00 0,94	r=-0,08 0,55	r=-0,18 0,42	r=-0,16 0,25
number oocytes				**r=0,68 <0,001**	**r=0,8 0,003**	**r=0,28 0,03**	r=-0,03 0,80	r=-0,03 0,83	r=0,06 0,64	r=0,02 0,89	r=0,04 0,91	r=-0,11 0,43
max estradiol					**r=0,25 0,05**	r=0,17 0,20	r=0,00 0,95	r=-0,09 0,50	r=0,04 0,75	r=0,06 0,66	r=-0,12 0,36	r=-0,26 0,07
vit B12 before						**r=0,88 <0,001**	r=-0,11 0,41	r=0,11 0,41	**r=0,44 <0,001**	**r=0,44 <0,001**	**r=-0,40 0,002**	**r=-0,45 0,001**
vit B12 after							r=-0,12 0,36	r=0,24 0,07	**r=0,42 0,001**	**r=0,49 <0,001**	**r=-0,39 0,003**	**r=-0,44 0,001**
8OH2dG/cr before								r=0,15 0,25	r=-0,07 0,58	r=0,00 0,96	r=-0,11 0,40	r=-0,10 0,48
8OH2dG/cr after									r=0,16 0,22	r=0,31 0,02	r=-0,23 0,08	r=-0,27 0,06
vit B9 before										**r=0,85 <0,001**	r=0,49 <0,001	r=-0,55 <0,001
vit B9 after											**r=-0,54 <0,001**	**r=-0,64 <0,001**
homocyst before												**r=0,87 <0,001**

There was a significant correlation (p<0.05) between the concentration of vitamin B12 before supplementation and both the number of oocytes (r=0.38) and maximum estradiol concentration (r=0.25), and between the concentration of vitamin B12 after supplement intake and the number of oocytes (r=0.28), but not between vitamin B12 concentration after intake and maximum estradiol concentration (r=0.17). Age was inversely correlated with the number of oocytes (r=-0.32) and maximum estradiol concentration (r=-0.27). The cycle number was also correlated with the maximum estradiol concentration (r=0.31). Finally, the ratio of 8OH- 2dG/creatinine in the urine was significantly correlated with the concentration of vitamin B9 after supplementation (r=0.31). However, the 8OH-2dG/creatinine after supplementation was not correlated with the 8OH-2dG/creatinine ratio before supplementation (r=0.15), nor with vitamin B9 before (r=0.16) nor with homocysteine before (r=-0.23).

### Methylation of the hTERT promoter region

A total of 219 samples have been assessed, including follicular fluid of two follicles of the left and two of the right ovaries, whenever available. For every sample the total degree of methylation of the 5 dinucleotides, CpG was quantified in the TERT promotor (Fig.1). The total methylation (CpG 1, 2, 3, 4 and 5 added) per sample was: mean 25.42% (SD: 13.27), median 22.46% (95% CI: 21.00-23.93).

**Figure 1:**
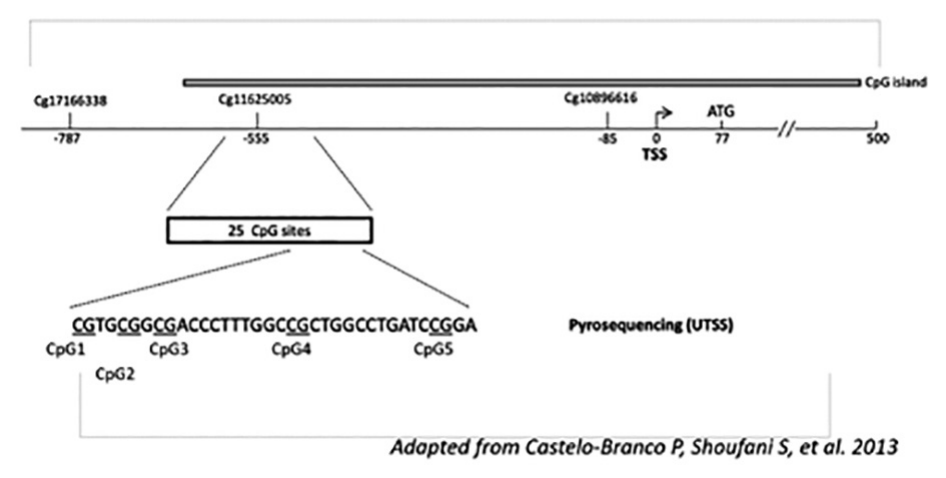
Schematic representation of the DNA fragment analysed by CpG pyrosequencing in the hTERT promoter region. Underlined are the 5 pyrosequenced CpG sites upstream of the TERT transcription start site (hence, UTSS), relative to CpG probe positions Cg17166338, Cg11625005 and Cg10896616 according to Illumina Infinium HumanMethylation450 arrays. DNA hypermethylation of the UTSS has been shown to associate with high TERT expression in cancer (Castelo-Branco et al., 2013).

There was no significant correlation between several samples taken from different follicles of the same patient.

Special attention was focussed on the degree of methylation of the CpG2 since this has previously been related to the risk of developing cancer (Comhaire et al., 2015; Castelo-Branco et al., 2013; Zhang et al., 2015). The degree of methylation of CpG2 per sample was as follows: geometric mean 7.68%, median 7.86% (CI: 7.24-9.14) in group 1 patients, geometric mean 6.85%, median 8.41% (CI: 6.86-9.58) in group 2 patients, and geometric mean 8.23, median 8.83% (CI: 6.99-10.14) in group 3 patients. The difference between group 2 and group 3 cases pooled with previous measurements was significant (P=0.039) (Fig.2).

**Figure 1:**
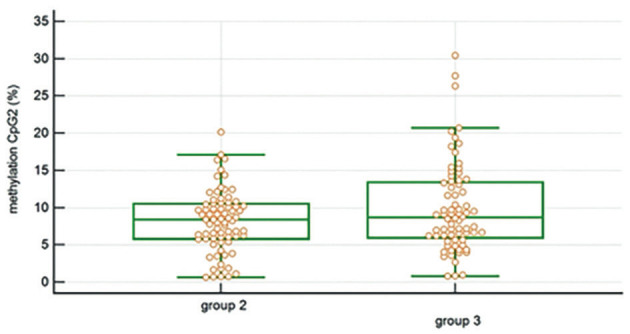
Schematic representation of the DNA fragment analysed by CpG pyrosequencing in the hTERT promoter region. Underlined are the 5 pyrosequenced CpG sites upstream of the TERT transcription start site (hence, UTSS), relative to CpG probe positions Cg17166338, Cg11625005 and Cg10896616 according to Illumina Infinium HumanMethylation450 arrays. DNA hypermethylation of the UTSS has been shown to associate with high TERT expression in cancer (Castelo-Branco et al., 2013).

### Multiple and logistic regression, ROC curve analysis, and comparison between groups.

In multiple regression analysis with stepwise elimination, the number of oocytes was found to be determined by the age of the woman and the concentration of vitamin B12 after supplementation (P= 0.003).

When analysing the probability of pregnancy per oocyte aspiration, (including fresh and frozen transfers) the logistic regression analysis selected age, cycle number, concentration of folic acid and the ratio of 8-OH-2dG over creatinine in early morning urine as independent variables with area under the ROC curve of 0.78, sensitivity of 81% and specificity of 68%, positive likelihood ratio of 2.5 and negative likelihood ratio of 0.37.

The total pregnancy rate per oocyte aspiration was only significantly higher in group 2 compared to group 3 (Fisher’s exact test: P=0.002) (Table II).

## Discussion

There were no significant differences between the participants of the three groups regarding age, BMI, number of previous cycles and the biological variables before treatment. Neither were there significant differences between the number of oocytes and the maximum estradiol concentration between groups. As expected, the latter variables were significantly correlated mutually.

The degree of methylation of the CpG2 of the hTERT promoter region of follicular cells was lower in both groups receiving the food supplement compared to the control group 3, but only the difference between groups 2 and 3 was significant. As such, larger cohorts may be needed to increase sensitivity/significance, and the selected hTERT promotor region could be extended to the TERT hyper-methylated oncological region (THOR), a 433-bp genomic region encompassing 52 CpG sites located immediately upstream of the TERT core promotor, associated with cancer-specific upregulation of TERT expression (Lee et al., 2019).

There was no correlation between the degrees of methylation of the hTERT promoter region in follicular fluids obtained from different follicles of the same patient. This relates to the well-known lack of synchronisation of follicular maturation, paracrine activity and metabolism during ovarian stimulation (Hillier, 2009).

The overall pregnancy rate after a fresh transfer is similar to that reported in another large controlled trial (Smith et al., 2018), and to that in the population treated in the same centre during the period of the trial (32%) (Comhaire and Decleer, 2020). The relatively low rate, especially in group 3 patients, may be related to characteristics of the patients included, which are known to negatively affect the probability of success. These include the rather high age (Spandorfer et al., 1998) of the participants (56% older than 30 years, 24% older than 35 years), their somewhat elevated BMI (Banker et al., 2017) (37% higher than 25, 19% higher than 30), and the fact that 68% of patients had gone through at least one unsuccessful IVF cycle before (Smith et al., 2015). The cumulative pregnancy rate (including fresh and frozen transfers) was significantly higher in group 2 than in group 3, with number needed to treat (NNT) of 2. When comparing groups 1 and 3, the NNT is 5, which is similar to that observed in previous placebo-controlled trial (Comhaire and Mahmoud, 2013).

Both the amount of oxidized DNA and the concentration of homocysteine were significantly decreased in group 2 patients taking the antioxidants astaxanthin and ubiquinone Q10 vitamins B9 and B12, as well as selenium. This observation highlights the complementary beneficial effect of adding selenomethionine, which also increased the pregnancy rate and reduced the degree of methylation of the second dinucleotide (CpG2) of the hTERT promoter region. This deserves further investigation by novel advanced SMRT sequencing methods, which can simultaneously detect multiple DNA methylation and oxidative damage DNA modifications (Nakano et al., 2017).

Selenium methionine is the organic compound that is the most appropriate form of selenium to be used as a food supplement, thanks to its excellent bioavailability and low toxicity. The amount of selenium intake in the populations of the UK, parts of Europe, New Zealand and certain regions of the US may be suboptimal. Inadequate selenium intake has been associated with impaired immunity and an increased risk of cancer. In fact, selenium supplementation may play a role in the prevention of cancer thanks to its effects on DNA-repair, apoptosis and the endocrine and immune systems (Spallholtz et al., 1990), as well as its antioxidant properties (Tinggi, 2008). In addition, selenium increases the level of P53, which is a large protein that corrects DNA damage caused by transition mutagenesis. As observed in the present trial, selenium may act as a chemo-preventive agent (Hughes et al., 2015; Kune and Watson, 2006; Zhao et al., 2006) by decreasing DNA-methylation through the reduction of DNA-(cytosine e-5)- methyl transferase (Mtase) (Fiala et al., 1998).

The weakness of the present trial is that it is performed in a single centre and includes a limited number of cases. However, the pragmatic approach has the merit of reflecting the “real life” situation as it occurs in centres of reproductive medicine, and its strength resides in the randomised prospective design (Mathes et al., 2018; 2019).

The findings have induced the development of a novel nutraceutical formulation (QALY 1+2 ® , Jonapharma, Elversele, Belgium) that may be recommended for preconception administration to all women who are to have IVF or intra-uterine insemination with ovarian stimulation (Oostingh et al., 2019). The nutraceutical may also benefit infertile patients suffering from polycystic ovary syndrome who present distinct changes in the proteome profile of endometrial tissue (Amjadi et al., 2018) and abnormal homocysteine metabolism (Li et al., 2018) related to oxidative stress, inflammation and insulin resistance (Legro et al., 2007). It is hypothesised that the preconception intake of this food supplement may reduce the risk of neoplastic diseases in the offspring, particularly by decreasing the degree of methylation of the hTERT promoter region. Larger multicentre controlled trials are needed to test this hypothesis.

## Conclusion

The study allows for several conclusions. The food supplementation with a specific nutraceutical creates an internal environment at the time of IVF pick-up and conception that would generally be considered favourable because of decreased oxidative DNA damage and the lower homocysteine concentration. The homocysteine concentration in blood has been considered representative of the concentration in the follicular fluid (Szymanski and Kazdepka- Zieminska, 2003; Berker et al., 2009). The finding of decreased methylation of the second dinucleotide CpG of the hTERT promoter region of follicular cells does reflect the situation of the oocytes and may possibly reduce the risk of neoplastic diseases in IVF offspring.

Though the higher pregnancy rate in women treated with the selenium-enriched nutraceutical needs to be certified in larger trials, the low NNT together with the minimal cost price of the nutraceutical should significantly reduce the cost per pregnancy (Comhaire and Decleer, 2011).

## Appendix

Composition of Fertility woman ® (registered by Nutriphyt Ltd, Beernem, Belgium): Lepidium meyenii 125mg ; Pinus maritima 50mg ; Acetyl-L-carnitine 50mg ; Co-enzyme Q10: 12.5mg ; Zincglycinate 3.75mg ; Astaxanthine 4mg ; Vitamin B6 1.5mg ; Vitamin B9 0.1mg ; Vitamin B12 0.75μg.

Two capsules of this combination per day to be taken together with two capsules per day of polyunsaturated omega 3 fatty acids (DHA and EPA 1000 mg).
